# Case Report: Congenital Pseudoacorea in an Ocular Axenfeld-Rieger Syndrome: What is it?

**DOI:** 10.46619/joccr.2023.6-1145

**Published:** 2023-03-28

**Authors:** Andrés Germán Alza

**Affiliations:** Private Eye Clinic Dr. Enrique Alza, Buenos Aires - Argentina

**Keywords:** Eye, Hidden pupil, Axenfeld-Rieger syndrome, Surgical management

## Abstract

**SUMMARY:**

This is a descriptive summary of the case of a patient with Axenfeld-Rieger syndrome associated with a congenital malformation of the iris and consequent pupillary morphological alteration of an atypical characteristic reported. This anomaly is unique in scientific literature and exhibits a peculiarity that we have called pseudoacorea: Hidden pupil. Other associated abnormal clinical findings were posterior embryotoxon, astigmatism, amblyopia, and exotropia. Diagnosis was achieved by instilling ocular mydriatics into the cul-de-sac that revealed this peculiarity. It is necessary to make a differential diagnosis with other pupillary pathologies such as corectopia, acorea and microcoria. Early detection of pathology and surgical management is necessary, since it would lead to a better visual prognosis for both amblyopia and strabismus.

**BACKGROUND:**

Among the malformations of the pupil, we can find polycoria (more than one pupil), dyscoria (abnormal pupil shape), corectopia (abnormal pupil position) and acorea (absence of pupil). In addition, morphologically normal pupils can denote other anomalies such as the microcoria described by Holth in 1923. Acorea is a rare anomaly, congenital or acquired, characterized by an absolute absence of the pupil both at rest and in mydriasis. In our case we prefer to differentiate it and name it pseudoacorea, since although there is a total absence of the pupil at rest thanks to the application of ocular mydriatics, a micropupil with discoric and corectopic characteristics is achieved. It is worth noting that we have not detected in the scientific literature any case described as the one that we will develop here.

**CONCLUSION:**

The case of a patient with Axenfeld-Rieger syndrome associated with a congenital malformation of the iris and consequent atypical pupillary morphological alteration is presented. This anomaly is unique in the scientific literature and presents a peculiarity that we have called pseudoacorea: Hidden pupil. Early detection of pathology and surgical management is necessary, since it would lead to a better visual prognosis for both amblyopia and strabismus.

## CASE REPORT

**Figure 1.**
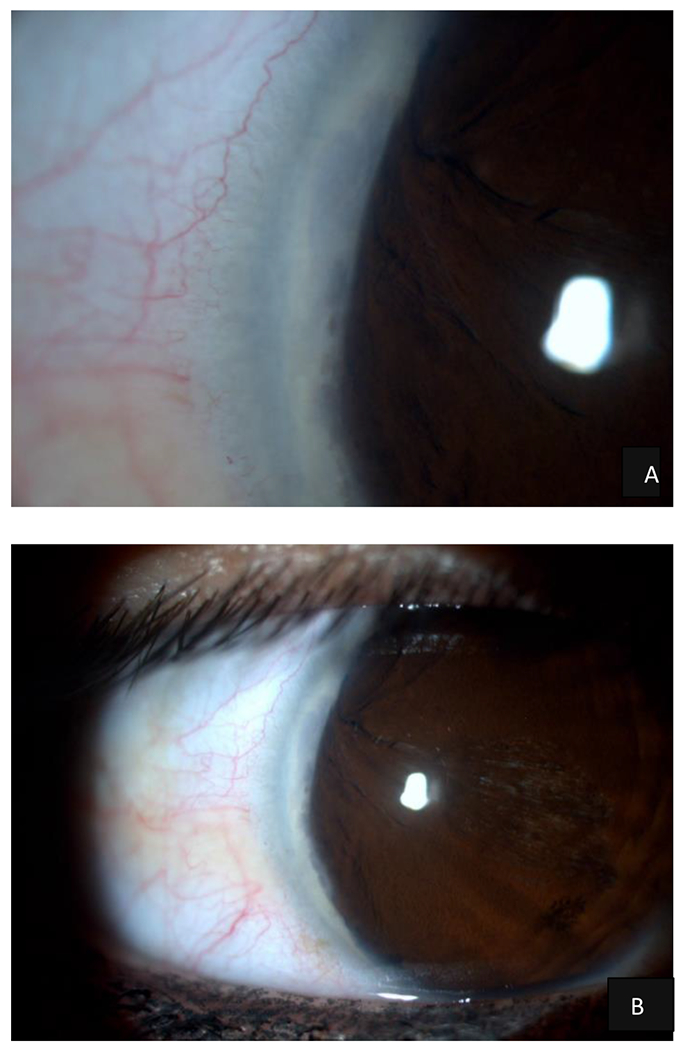

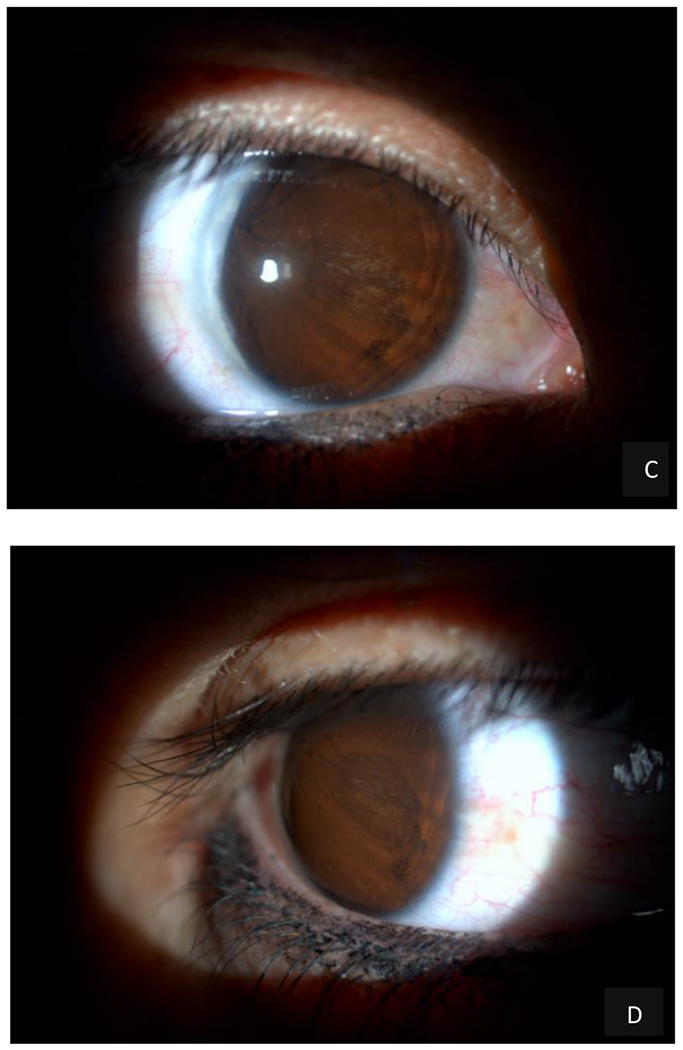
Slit-lamp biomicroscopy of the RE: The peripheral cornea was associated with a whitish, arcuate, and concentric ridge parallel to the sclerocorneal limbus, with well-defined borders at a temporal level from 6 to 12 hours that affected the entire corneal thickness, belonging to a later embryotoxon. Thick iris with displaced crypts, absence of vascular rings, and depigmentation in the visual axis, the latter possibly being the result of failed laser treatment. A - B - C - D Source: Own creation C - Source: Own creation taken from Alza A and Galletto E (2022) Retroiridian pupilloplasty. Clinical and Experimental Ophthalmology 15(1): e40i-e47i.

**Figure 2:**
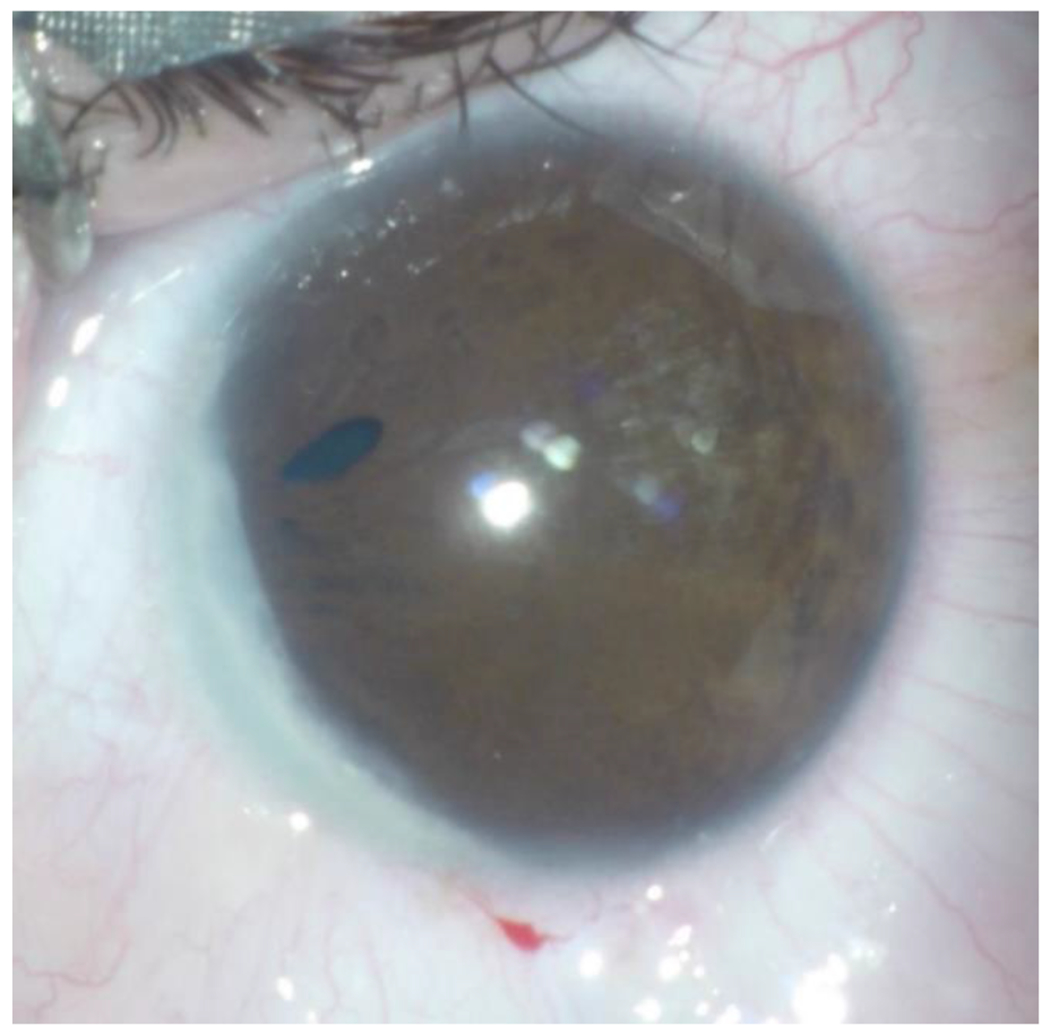
Slit-lamp biomicroscopy of the RE: After the application of mydriatic eye drops in the fornix, a corectopia towards temporality of a discoric micropupil in the form of a peripheral horizontal teardrop is detected. Source: Own creation.

**Figure 3:**
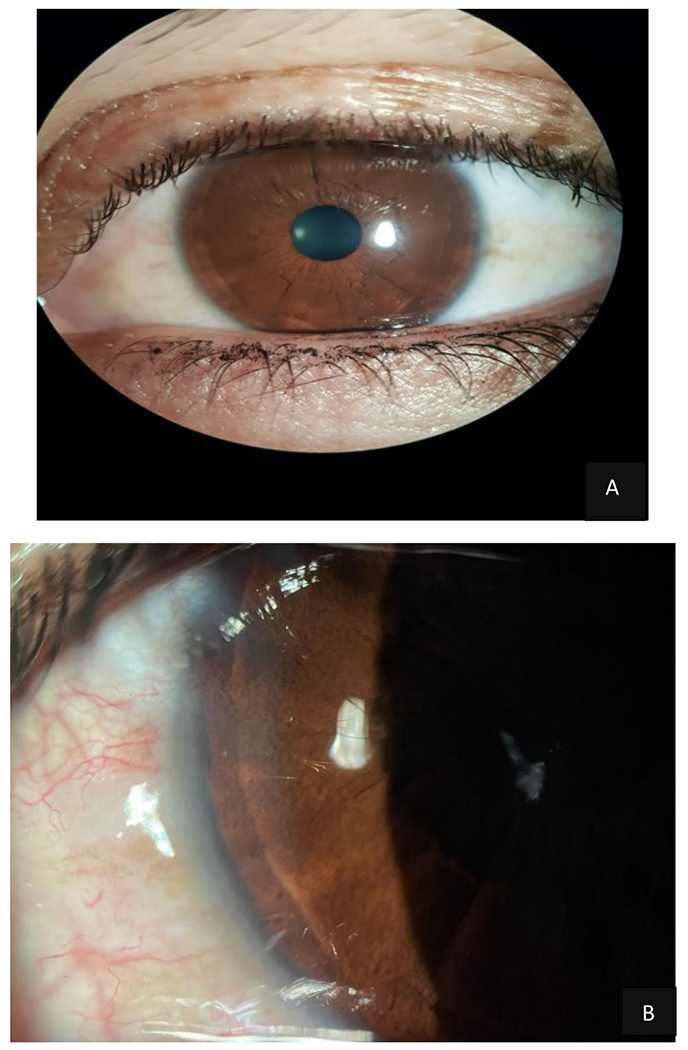
Slit-lamp biomicroscopy of the LE: Normal in appearance and only with the presence of a very poorly defined posterior embryotoxon that affected the corneal periphery parallel to the sclerocorneal limbus. Source: A - B Own creation.

**Figure 4:**
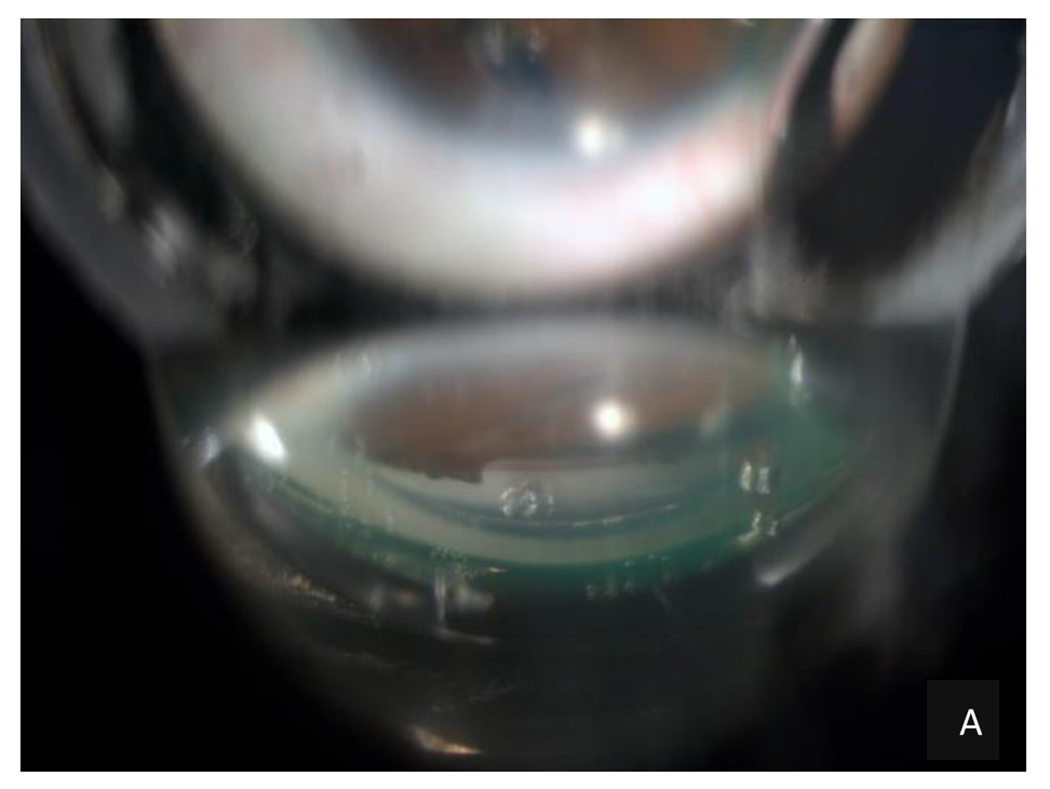

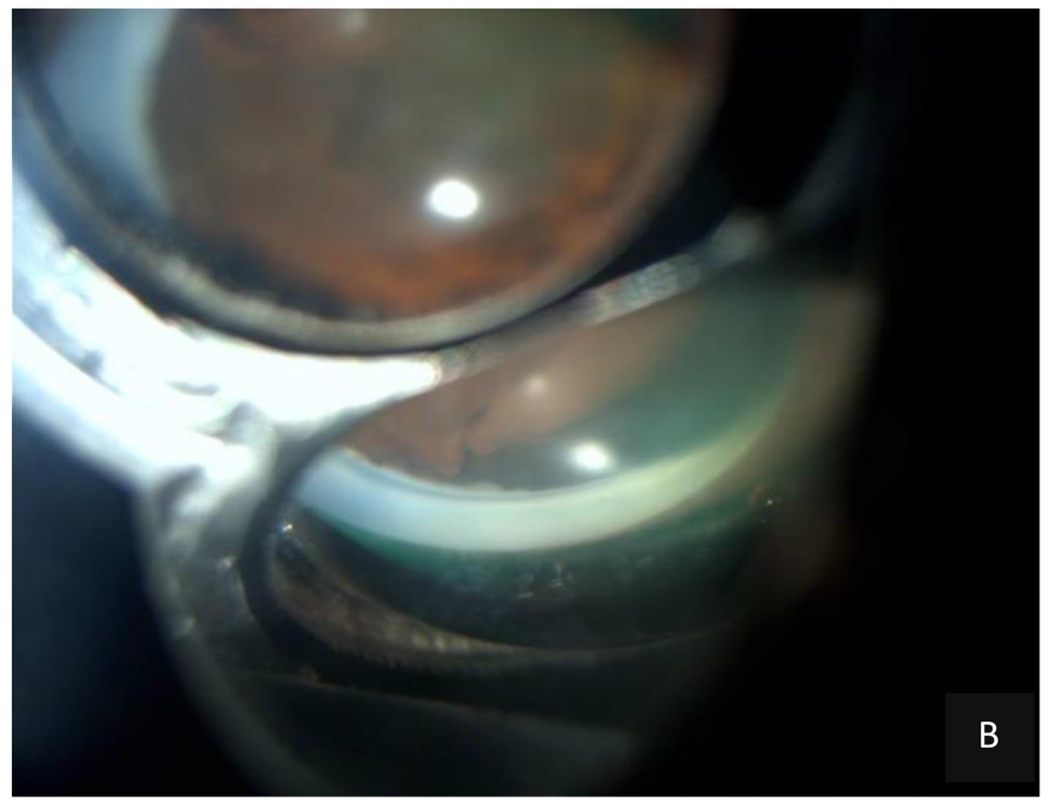
Gonioscopy of the RE: Schwalbe’s line dysgenesis associated with peripheral anterior synechiae that produced traction of the iris with a marked displacement that hid the pupil giving the characteristic appearance of pseudoacorea was evidenced. Source: A Own creation taken from Alza A and Galletto E (2022) Retroiridian pupilloplasty. Clinical and Experimental Ophthalmology 15(1): e40i-e47i.- B Own creation.

**Figure 5:**
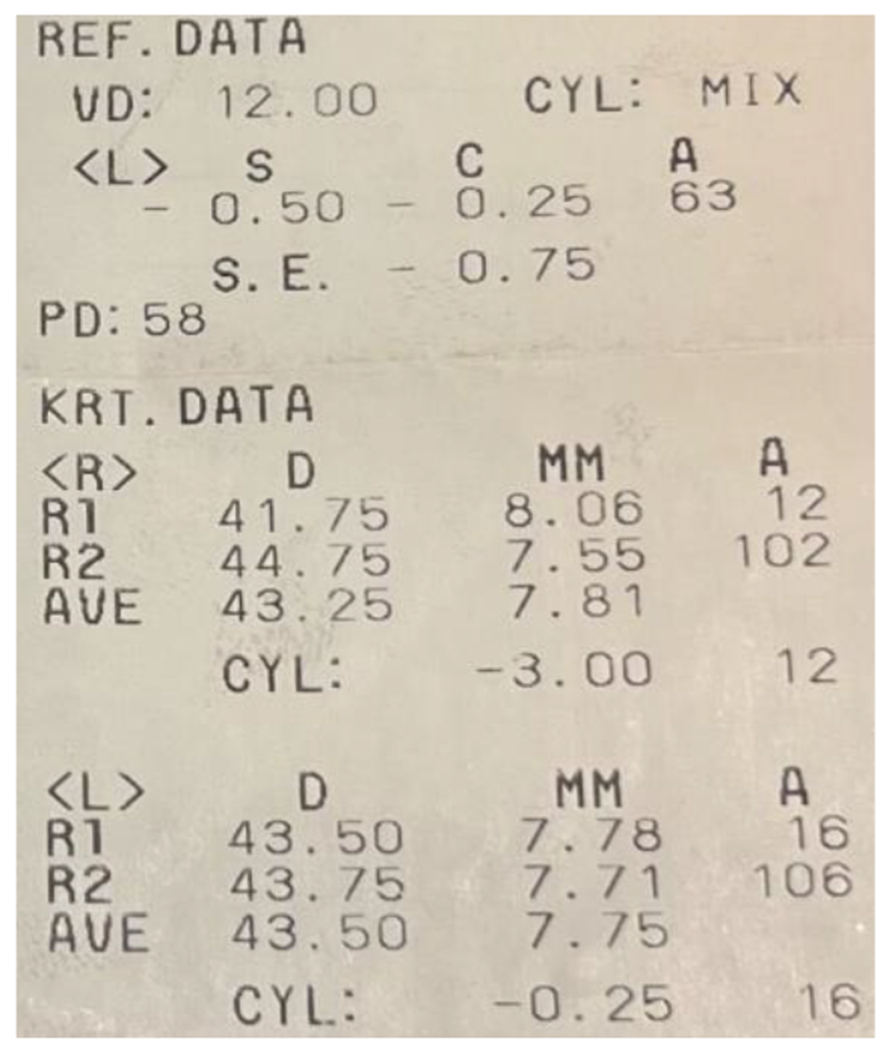
(RE - LE) The refraction test with an autorefractometer gave a raised corneal cylinder of −3.00 diopters in the RE, which was only of −0.25 in the LE. Source: Own creation.

**Figure 6:**
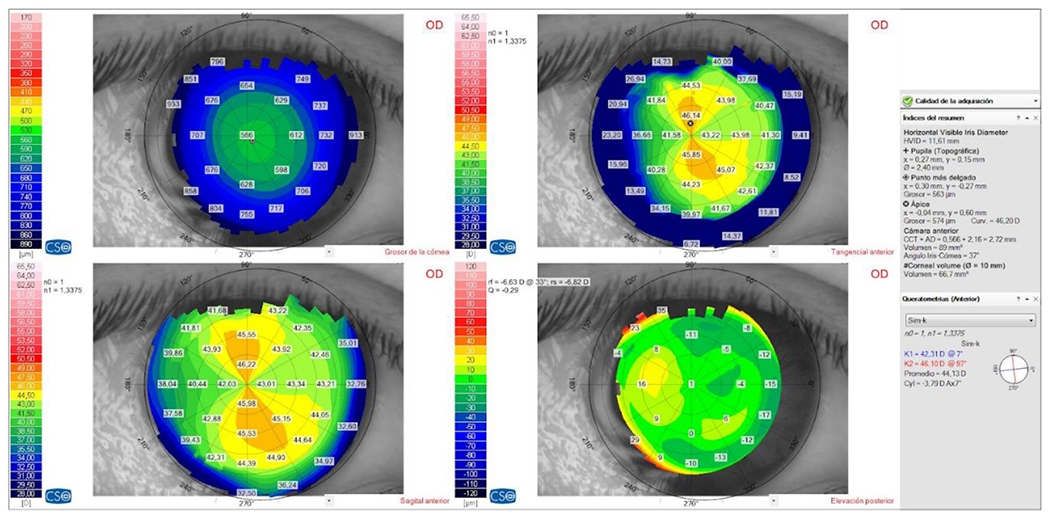

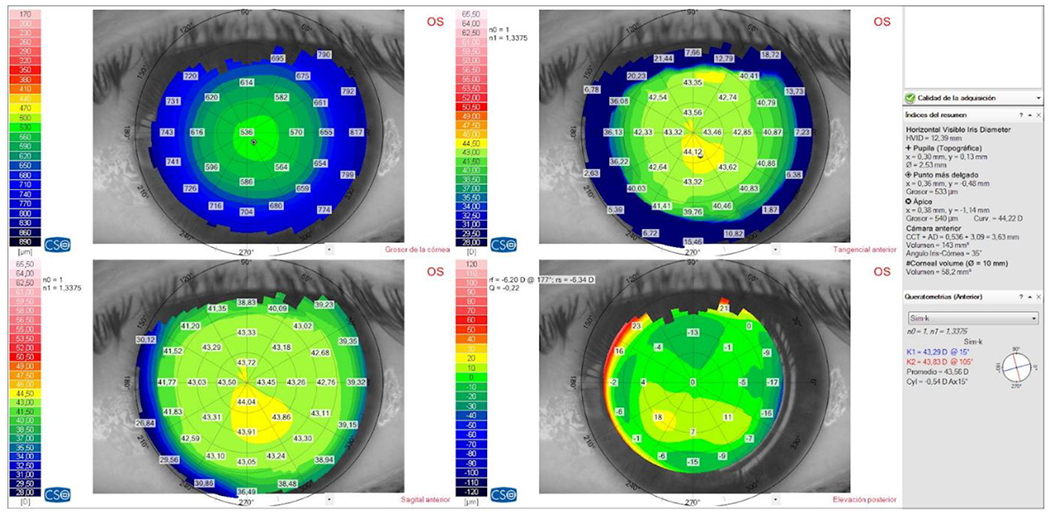
RE (OD) - LE (OS) Corneal surface topography and pachymetry within the normal range. Source: Own creation.

**Figure 7:**
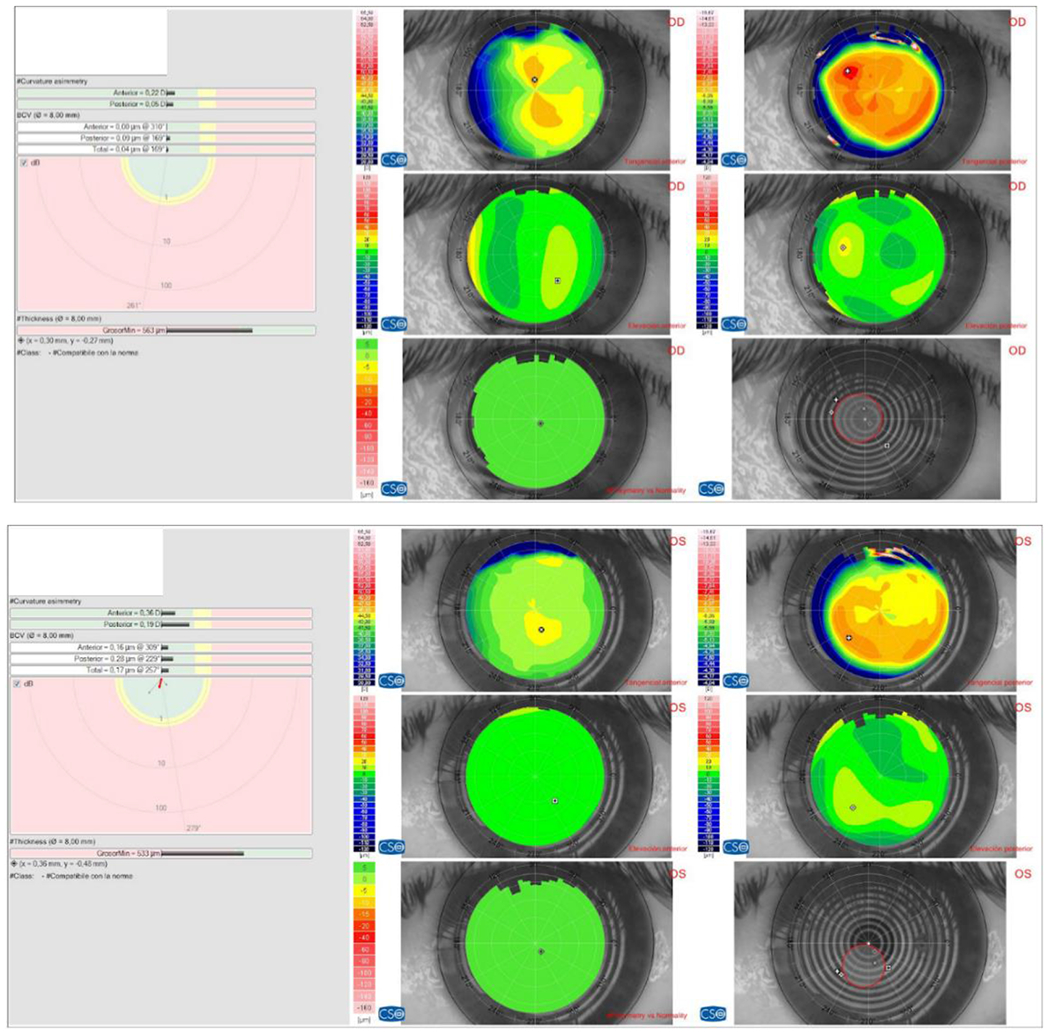
RE (OD) - LE (OS) Posterior elevation topography within the normal range. Source: Own creation.

**Figure 8:**
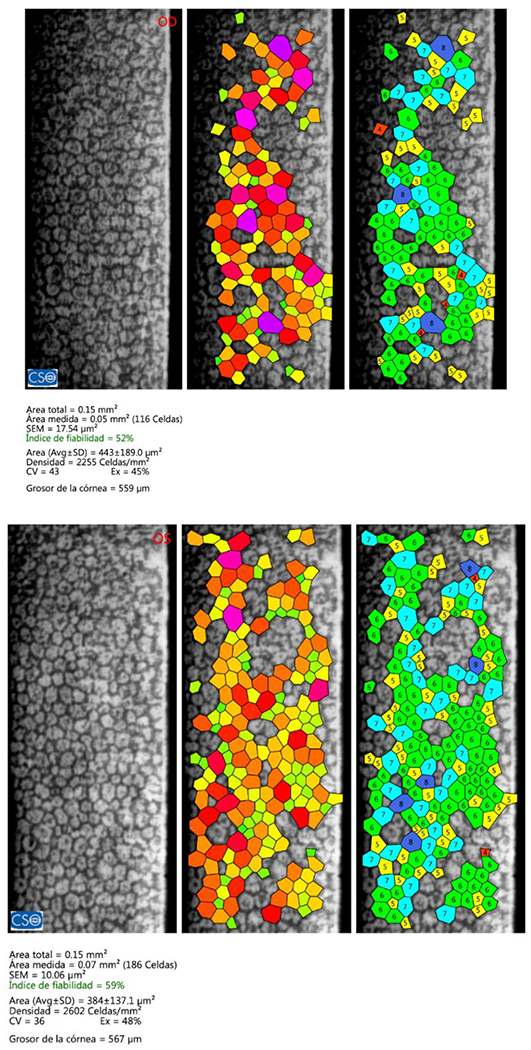
RE (OD) - LE (OS)Endothelial count examination within the normal range. Source: Own creation.

**Figure 9:**
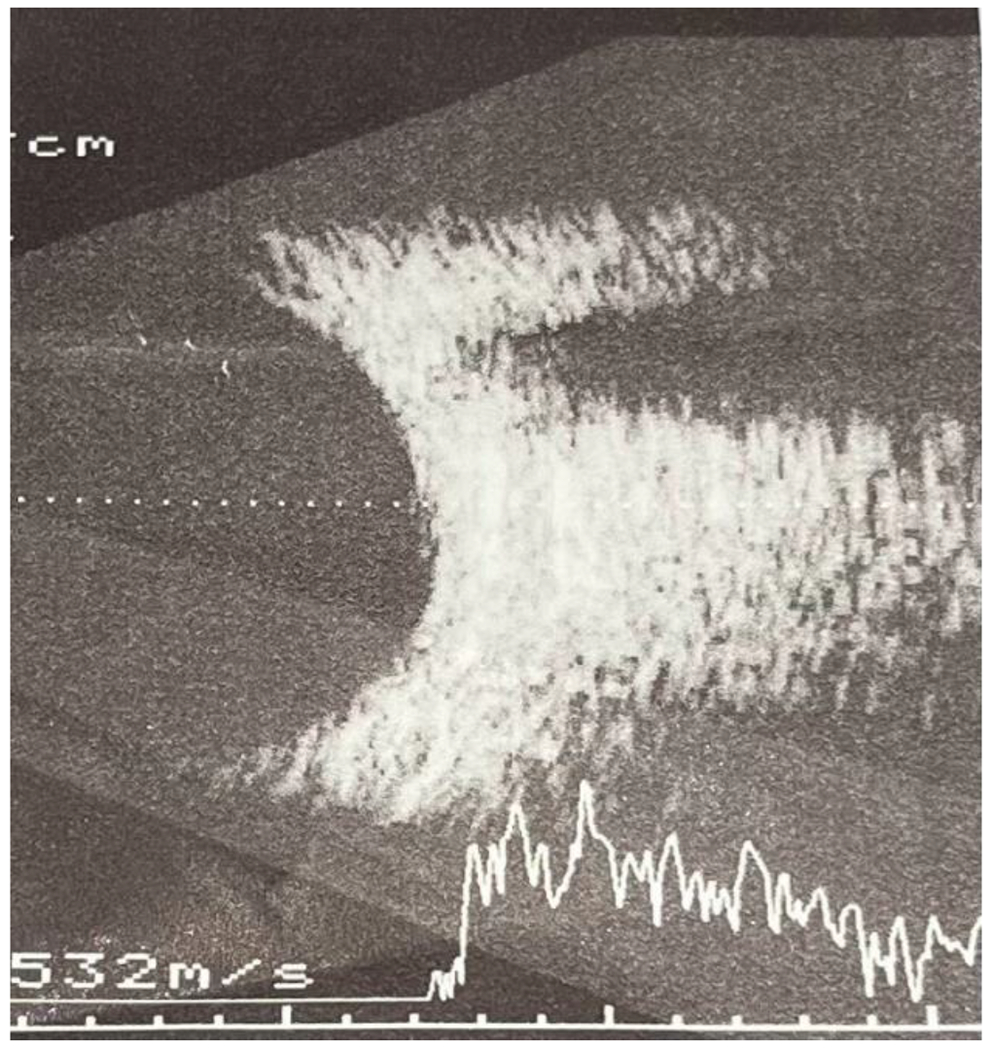
Ocular ultrasonography of the RE within the normal range. Source: Own creation.

**Figure 10:**
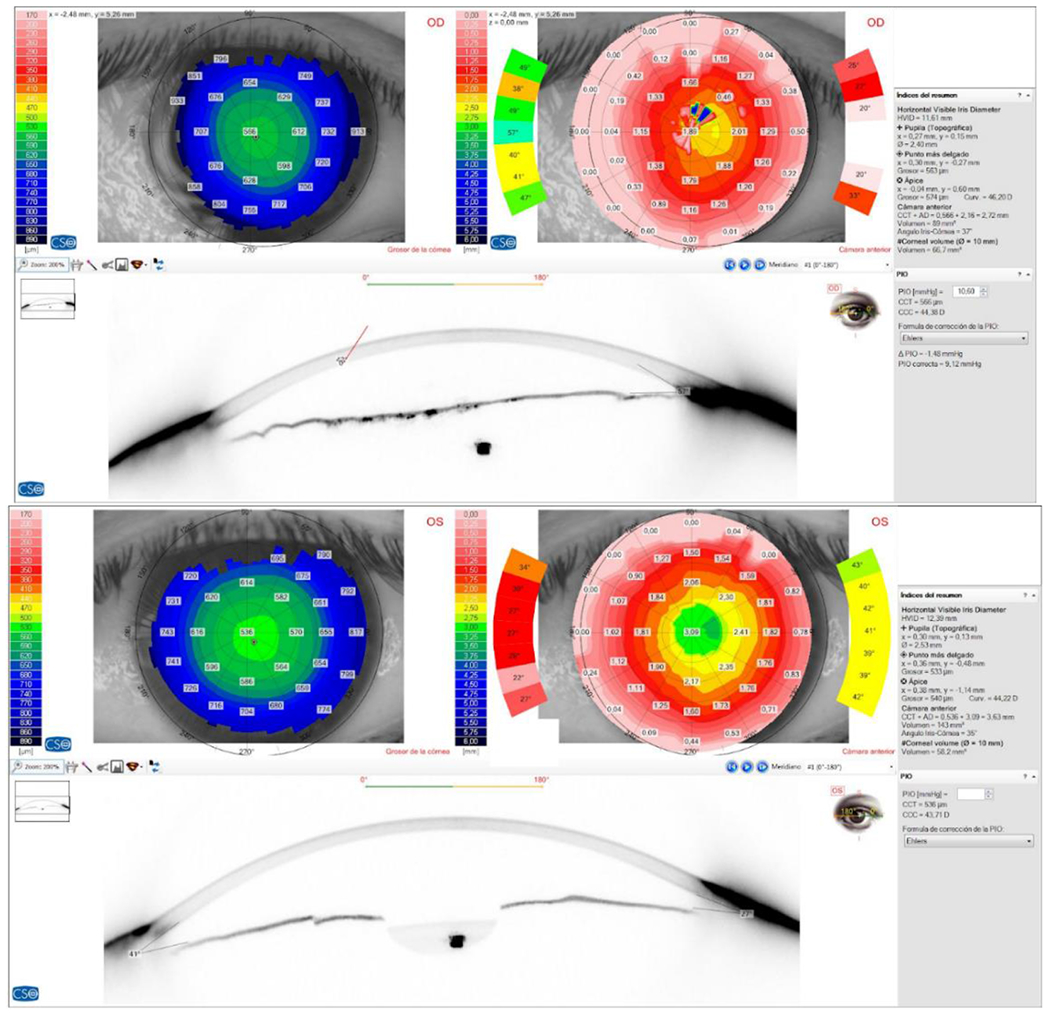
RE (OD) - LE (OS) The study of corneal topography with a Scheimpflug camera revealed an absence of pupil in the RE and a smaller horizontal visible iris diameter than in the LE where the parameters were normal. Source: Own creation.

**Figure 11:**
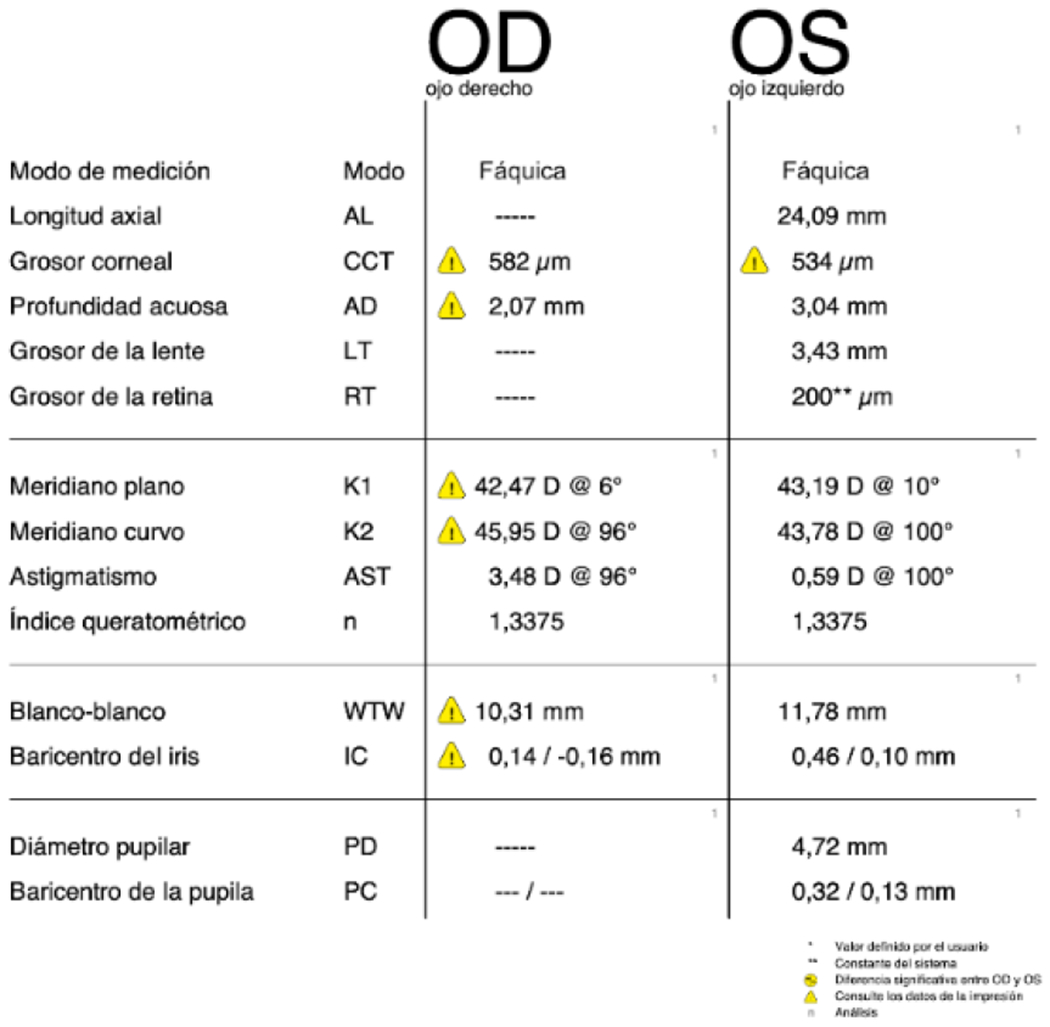
RE (OD) - LE (OS) Non-contact biometry of the RE was impossible to carry out due to the absence of the pupil, and an axial length equivalent to myopia was determined in the LE. Source: Own creation.

**Figure 12:**
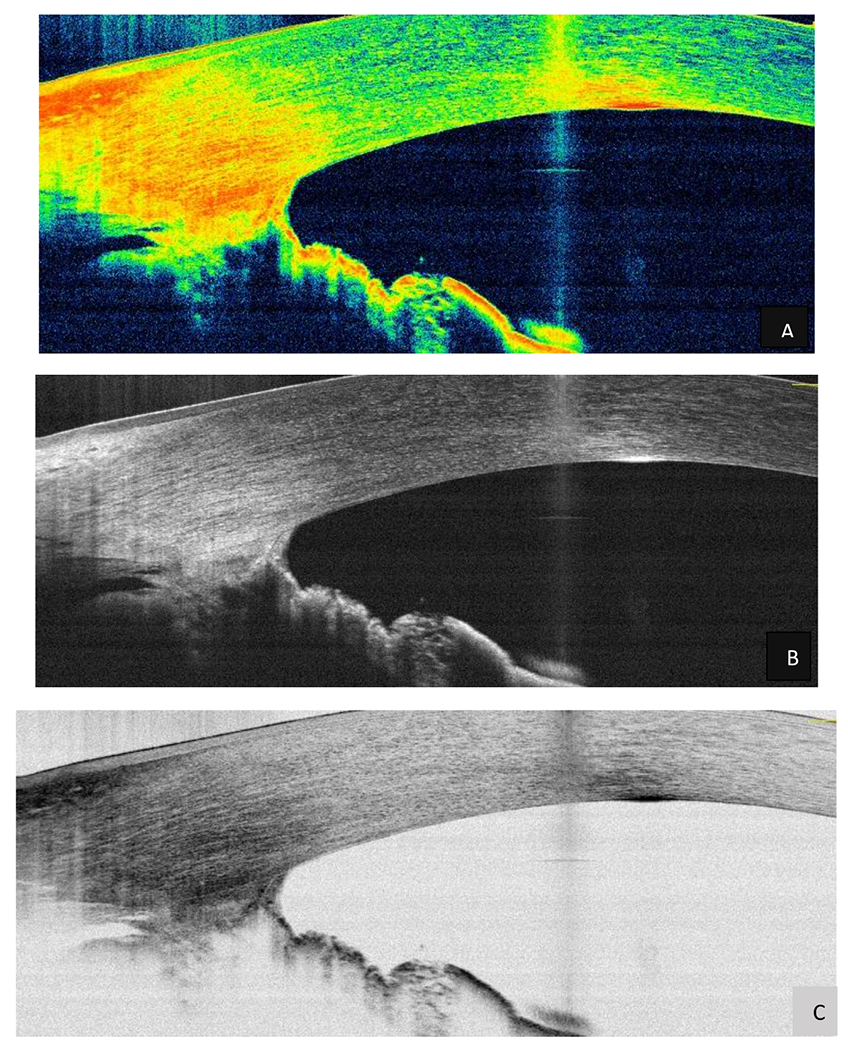
Optical tomography of the anterior pole of the RE shows ocular dysgenesis with the appearance of a posterior embryotoxon and adhesion bands to the peripheral processes of the iris, creating iridocorneal junction bridges. A - B - C Source: Own creation.

**Figure 13:**
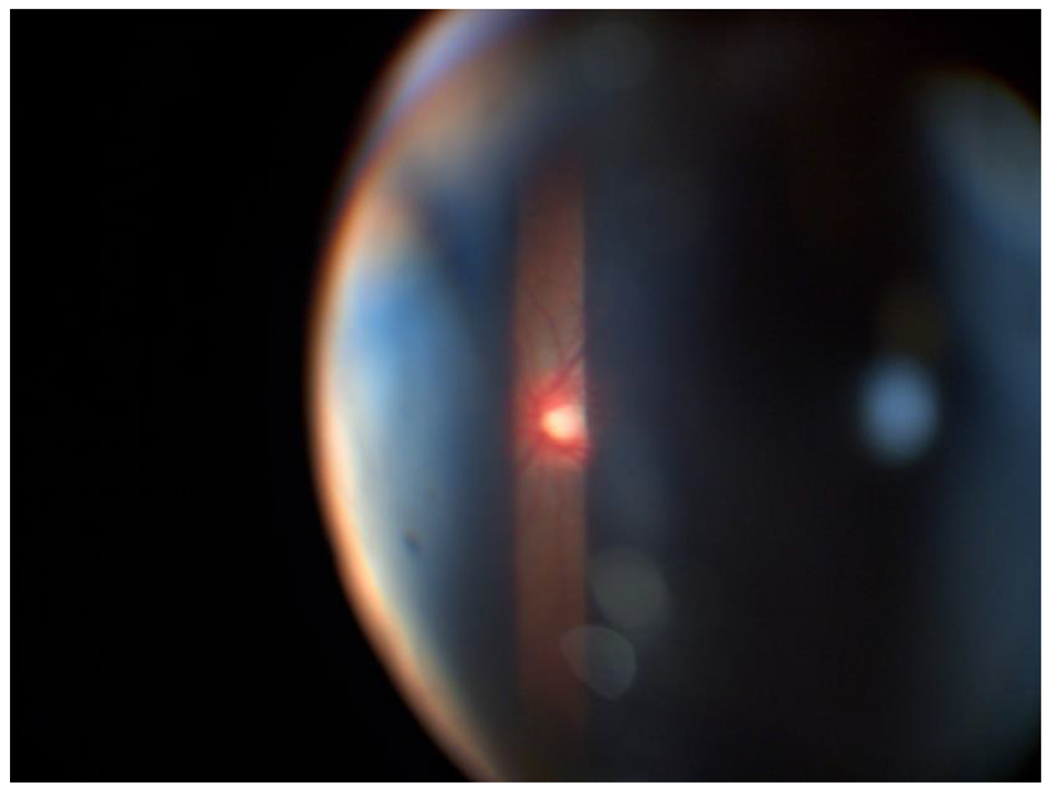
Fundus of the RE after the surgical technique called retroiridian pupilloplasty. Source: Own creation.

**Figure 14:**
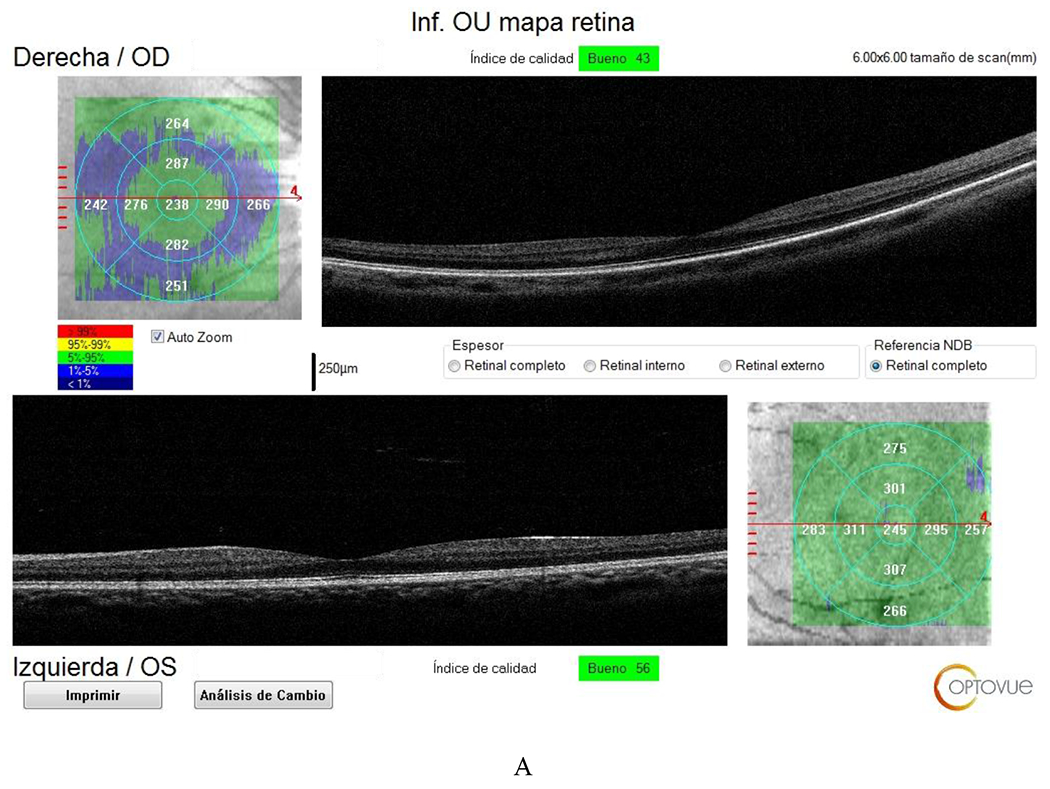

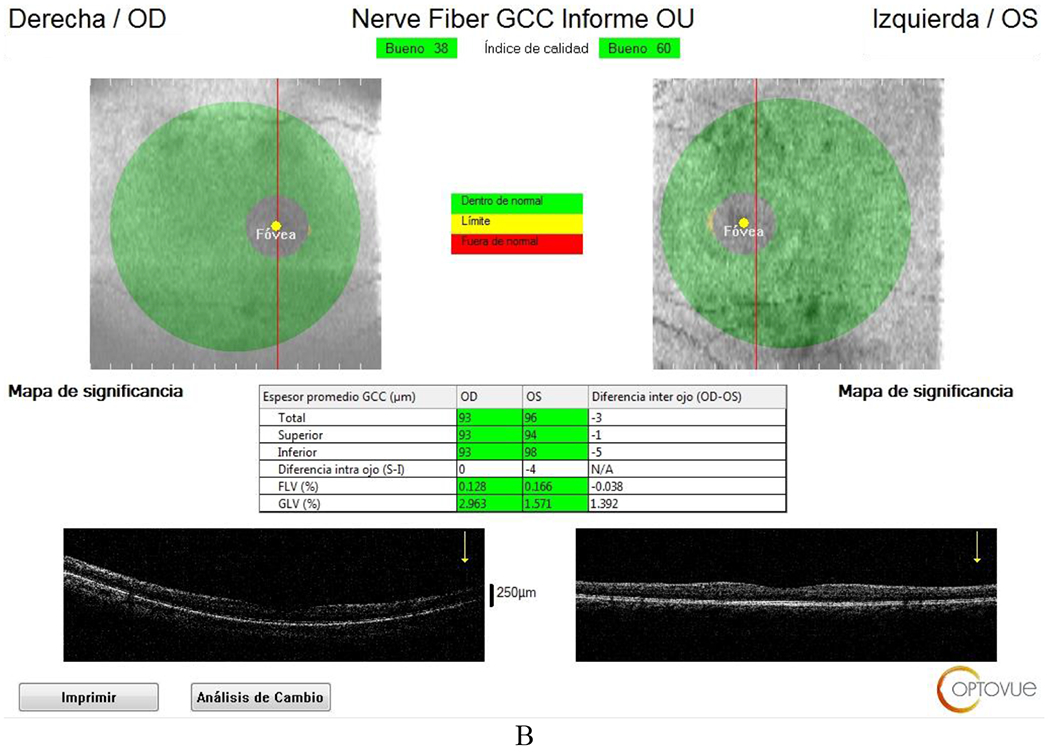
RE (OD) - LE (OS) Optical coherence tomography: Macula of the RE resulting in atrophy of the thickness of nerve fibers and LE are conserved. The retinal ganglion cells in the RE - LE are conserved. Source: A - B own creation.

**Figure 15:**
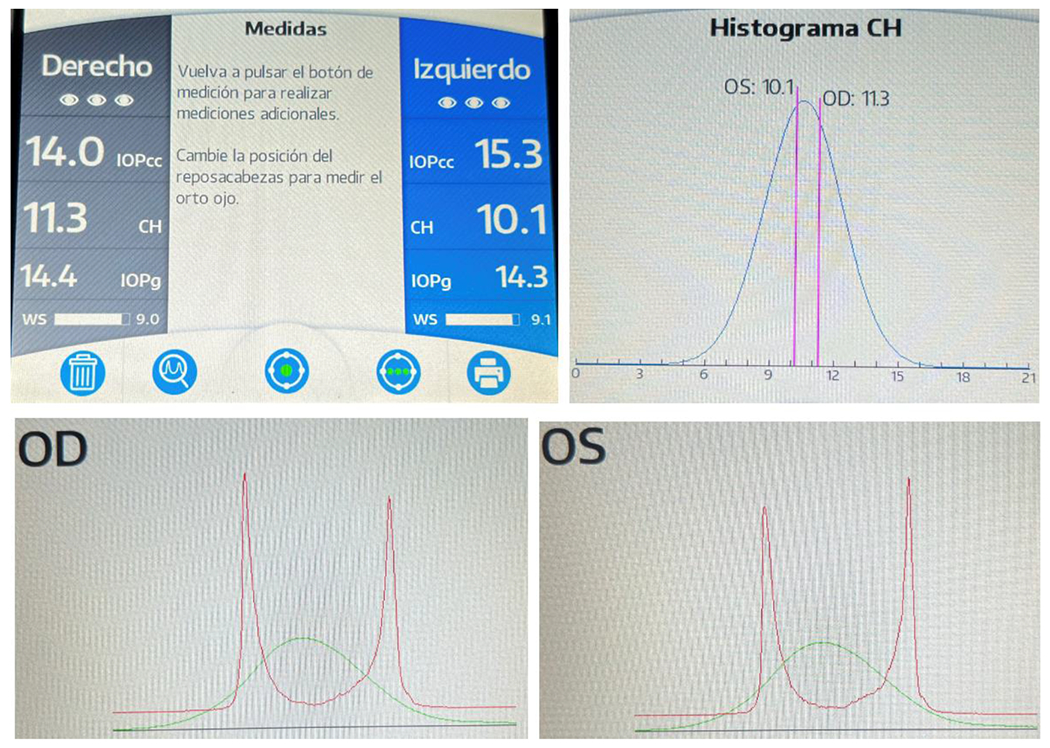
RE (OD) - LE (OS) ORA a IOPcc was 14 mmHg and 15.3 mmHg and bilateral HC was 11.3 and 10.1, within normal limits. Source: Own creation.

A 28-years old female Argentinian patient, and with Axenfeld-Rieger syndrome, who attended the ophthalmologic consultation reporting never having had a pupil in her right eye (RE), evident when looking in the mirror, and her left eye (LE) being of a normal appearance. As a personal history, she reported a phototherapy treatment resulting from neonatal jaundice as well as isolated episodes of seizures associated with febrile symptoms. She did not present a hereditary family history of any kind. At the time of the consultation, she expressed deep concern about her personal aesthetics as a result of her absence of pupils, a situation that brought her to a hospital ophthalmologic service where they tried to perform a laser pupilloplasty on her that was ineffective. The visual acuity test using the Snellen chart for distant vision showed only light vision in her RE, and 20/20 for her LE. Strabismus examination revealed a slight exotropia. On slit-lamp biomicroscopy examination of the RE, the peripheral cornea exhibited a whitish, arcuate, and concentric ridge parallel to the sclerocorneal limbus, with well-defined edges at the 6-to-12-hour temporal level that affected the entire corneal thickness, belonging to a posterior embryotoxon associated with a completely absent pupil (Figure 1). After the application of mydriatic eye drops in the fornix of the sac, a temporal corectopia of a discoric micropupil in the form of a peripheral horizontal teardrop was detected (Figure 2) and in the LE only the presence of a very poorly defined posterior embryotoxon that affected the corneal periphery (Figure 3). The ocular pressure with the Goldmann applanation tonometer was 17 mmHg and 18 mmHg respectively. Examination with the Goldmann lens for gonioscopy revealed dysgenesis of Schwalbe’s line associated with peripheral anterior synechiae in the RE that produced traction of the iris with a marked displacement that hid the pupil, giving the characteristic appearance of pseudoacorea (Figure 4) and the LE it only showed a tenuous posterior embryotoxon. The evaluation of the fundus of the eyes was impossible for the RE due to the hidden pupil and in the LE it did not show alterations. Given the findings described above, it was decided to request complementary studies for a more detailed evaluation. The refraction test with an autorefractometer gave a raised corneal cylinder of −3.00 diopters in the RE, which was only of −0.25 in the LE. (Figure 5). Corneal surface topography and pachymetry (Figure 6), Posterior elevation topography (Figure 7), endothelial count examination (Figure 8) and ocular ultrasonography (Figure 9), yielded results for both eyes within the normal range. The study of corneal topography with a Scheimpflug camera revealed an absence of pupil in the RE and a smaller horizontal visible iris diameter than in the LE where the parameters were normal (Figure 10). Non-contact biometry of the RE was impossible to carry out due to the absence of the pupil, and an axial length equivalent to myopia was determined in the LE (Figure 11). Optical tomography of the anterior pole shows ocular dysgenesis in the RE with the appearance of a posterior embryotoxon and adhesion bands to the peripheral processes of the iris, creating iridocorneal junction bridges (Figure 12). Once the ophthalmological evaluation was completed, it was decided to schedule a surgical treatment known as phakic retroiridian pupilloplasty to generate a neopupil. Subsequently, it was decided to complete the ophthalmological evaluation of the RE by performing an eye fundus that was apparently normal (Figure 13) and the optical tomography of the posterior pole shows macular atrophy of the thickness of the nerve fibers in the RE and LE within the normal range. The retinal ganglion cells in the RE - LE are preserved (Figure 14). The Ocular Response Analyzer (ORA) measured a compensated corneal intraocular pressure (IOPcc) of 14 mmHg and 15.3 (RE-LE). Bilateral corneal hysteresis (HC) was within normal limits (Figure 15).

## DISCUSSION AND CONCLUSION

Acorea is an iris anomaly with very few cases described and is characterized by the absolute absence of the pupillary opening, unlike pseudoacorea where the absence is relative, that is, “the pupil is hidden” [1,2]. Iridocorneal dysgenesis is the result of a failure to differentiate embryonic tissues from the anterior segment [3]. Within this group we find the Axenfeld-Rieger Syndrome (ARS) whose ocular clinical picture is posterior embryotoxon, peripheral anterior synechiae, iris atrophy, polychoria and corectopia associated with a high risk of glaucoma and systemic alterations such as defects on bone, facial and/or dental structures [4,5]. To explain the ocular alterations, there is a theory of the mechanism postulated by Shields which implies an arrest in the migration of neural crest cells towards the third trimester of gestation, which leads to the persistence of primordial endothelial tissue in the iris and anterior chamber angle. Contraction of this tissue after birth leads to the progressive changes seen in some patients. This primordial endothelium also generates an excessive and atypical basement membrane, especially near the limbal corneal junction, which explains the prominent Schwalbe line. In the case of secondary glaucoma, it would be the consequence of dysgenesis in the chamber sinus [6]. ARS is a rare disease that affects the eye bilaterally and asymmetrically with an estimated prevalence of 1/100,000 people, with no gender predilection and autosomal dominant hereditary characteristic with complete penetrance of variable expressivity [7]. The objective of this work is to present a rare case of ARS with a clinical sign that we will define as congenital pseudoacorea due to a total absence of the pupil at rest but after instillation of mydriatic drops of 5% phenylephrine +0.5% tropicamide in the bottom of the sac, obtaining a discoric micropupil in the shape of a lying teardrop, peripheral to the temporal. We have not detected previous similar reports to know the causes. This case in itself should be differentiated from size alterations such as microchoria, the latter is characterized by a pinpoint or pinhole pupil less than 2 mm in diameter both at rest and in mydriasis due to hypoplasia of the iris with little pupillary play [8]. The fact that the pupil is hidden but not absent would slightly improve the visual prognosis of patients, so it is essential in these cases to perform a pupilloplasty at an early age to avoid profound amblyopia and strabismus that is resistant to treatment. We developed an unprecedented surgical technique called retroiridian pupilloplasty whose objective was to create a new pupil both in a resting situation and in mydriasis [9]. In this case, the patient presented at an adult age, obscuring his visual prognosis but resolving the aesthetic aspect since, although the amblyopia was profound, her strabismus was not so great.

## Data Availability

The datasets during and/or analyzed during the current study available from the corresponding author on reasonable request.

## ABBREVIATIONS

RE: Right Eye
LE: Left Eye
ARS: Axenfeld-Rieger Syndrome
